# A modified action framework to develop and evaluate academic-policy engagement interventions

**DOI:** 10.1186/s13012-024-01359-7

**Published:** 2024-04-12

**Authors:** Petra Mäkelä, Annette Boaz, Kathryn Oliver

**Affiliations:** 1 Department of Health Services Research and Policy, Faculty of Public Health and Policy, London School of Hygiene and Tropical Medicine, 15-17 Tavistock Place, Kings Cross, London, WC1H 9SH UK; 2 Health and Social Care Workforce Research Unit, The Policy Institute, Virginia Woolf Building, Kings College London, 22 Kingsway, London, WC2B 6LE UK

**Keywords:** Evidence-informed policy, Academic-policy engagement, Framework modification

## Abstract

**Background:**

There has been a proliferation of frameworks with a common goal of bridging the gap between evidence, policy, and practice, but few aim to specifically guide evaluations of academic-policy engagement. We present the modification of an action framework for the purpose of selecting, developing and evaluating interventions for academic-policy engagement.

**Methods:**

We build on the conceptual work of an existing framework known as SPIRIT (Supporting Policy In Health with Research: an Intervention Trial), developed for the evaluation of strategies intended to increase the use of research in health policy. Our aim was to modify SPIRIT, (i) to be applicable beyond health policy contexts, for example encompassing social, environmental, and economic policy impacts and (ii) to address broader dynamics of academic-policy engagement. We used an iterative approach through literature reviews and consultation with multiple stakeholders from Higher Education Institutions (HEIs) and policy professionals working at different levels of government and across geographical contexts in England, alongside our evaluation activities in the Capabilities in Academic Policy Engagement (CAPE) programme.

**Results:**

Our modifications expand upon Redman et al.’s original framework, for example adding a domain of ‘Impacts and Sustainability’ to capture continued activities required in the achievement of desirable outcomes. The modified framework fulfils the criteria for a useful action framework, having a clear purpose, being informed by existing understandings, being capable of guiding targeted interventions, and providing a structure to build further knowledge.

**Conclusion:**

The modified SPIRIT framework is designed to be meaningful and accessible for people working across varied contexts in the evidence-policy ecosystem. It has potential applications in how academic-policy engagement interventions might be developed, evaluated, facilitated and improved, to ultimately support the use of evidence in decision-making.

Contributions to the literature

- There has been a proliferation of theories, models and frameworks relating to translation of research into practice. Few specifically relate to engagement between academia and policy.
- Challenges of evidence-informed policy-making are receiving increasing attention globally. There is a growing number of academic-policy engagement interventions but a lack of published evaluations.
- This article contributes a modified action framework that can be used to guide how academic-policy engagement interventions might be developed, evaluated, facilitated, and improved, to support the use of evidence in policy decision-making.
- Our contribution demonstrates the potential for modification of existing, useful frameworks instead of creating brand-new frameworks. It provides an exemplar for others who are considering when and how to modify existing frameworks to address new or expanded purposes while respecting the conceptual underpinnings of the original work.

## Background

Academic-policy engagement refers to ways that Higher Education Institutions (HEIs) and their staff engage with institutions responsible for policy at national, regional, county or local levels. Academic-policy engagement is intended to support the use of evidence in decision-making and in turn, improve its effectiveness, and inform the identification of barriers and facilitators in policy implementation [1–3]. Challenges of evidence-informed policy-making are receiving increasing attention globally, including the implications of differences in cultural norms and mechanisms across national contexts [4, 5]. Although challenges faced by researchers and policy-makers have been well documented [6, 7], there has been less focus on actions at the engagement interface. Pragmatic guidance for the development, evaluation or comparison of structured responses to the challenges of academic-policy engagement is currently lacking [8, 9].

Academic-policy engagement exists along a continuum of approaches from linear (pushing evidence out from academia or pulling evidence into policy), relational (promoting mutual understandings and partnerships), and systems approaches (addressing identified barriers and facilitators) [4]. Each approach is underpinned by sets of beliefs, assumptions and expectations, and each raises questions for implementation and evaluation. Little is known about which academic-policy engagement interventions work in which settings, with scarce empirical evidence to inform decisions about which interventions to use, when, with whom, or why, and how organisational contexts can affect motivation and capabilities for such engagement [10]. A deeper understanding through the evaluation of engagement interventions will help to identify inhibitory and facilitatory factors, which may or may not transfer across contexts [11].

The intellectual technologies [12] of implementation science have proliferated in recent decades, including models, frameworks and theories that address research translation and acknowledge difficulties in closing the gap between research, policy and practice [13]. Frameworks may serve overlapping purposes of describing or guiding processes of translating knowledge into practice (e.g. the Quality Implementation Framework [14]); or helping to explain influences on implementation outcomes (e.g. the Theoretical Domains Framework [15]); or guiding evaluation (e.g. the RE-AIM framework [16, 17]. Frameworks can offer an efficient way to look across diverse settings and to identify implementation differences [18, 19]. However, the abundance of options raises its own challenges when seeking a framework for a particular purpose, and the use of a framework may mean that more weight is placed on certain aspects, leading to a partial understanding [13, 17].

‘Action frameworks’ are predictive models that intend to organise existing knowledge and enable a logical approach for the selection, implementation and evaluation of intervention strategies, thereby facilitating the expansion of that knowledge [20]. They can guide change by informing and clarifying practical steps to follow. As flexible entities, they can be adapted to accommodate new purposes. Framework modification may include the addition of constructs or changes in language to expand applicability to a broader range of settings [21].

We sought to identify one organising framework for evaluation activities in the Capabilities in Academic-Policy Engagement (CAPE) programme (2021–2023), funded by Research England. The CAPE programme aimed to understand how best to support effective and sustained engagement between academics and policy professionals across the higher education sector in England [22]. We first searched the literature and identified an action framework that was originally developed between 2011 and 2013, to underpin a trial known as SPIRIT (Supporting Policy In health with Research: an Intervention Trial) [20, 23]. This trial evaluated strategies intended to increase the use of research in health policy and to identify modifiable points for intervention.

We selected the SPIRIT framework due to its potential suitability as an initial ‘road map’ for our evaluation of academic-policy interventions in the CAPE programme. The key elements of the original framework are catalysts, organisational capacity, engagement actions, and research use. We wished to build on the framework’s embedded conceptual work, derived from literature reviews and semi-structured interviews, to identify policymakers’ views on factors that assist policy agencies’ use of research [20]. The SPIRIT framework developers defined its “locus for change” as the policy organisation ( [20], p. 151). They proposed that it could offer the beginning of a process to identify and test pathways in policy agencies’ use of evidence.

Our goal was to modify SPIRIT to accommodate a different locus for change: the engagement interface between academia and policy. Instead of imagining a linear process in which knowledge comes from researchers and is transmitted to policy professionals, we intended to extend the framework to multidirectional relational and system interfaces. We wished to include processes and influences at individual, organisational and system levels, to be relevant for HEIs and their staff, policy bodies and professionals, funders of engagement activities, and facilitatory bodies. Ultimately, we seek to address a gap in understanding how engagement strategies work, for whom, how they are facilitated, and to improve the evaluation of academic-policy engagement.

### Aim

We aimed to produce a conceptually guided action framework to enable systematic evaluation of interventions intending to support academic-policy engagement.

## Methods

We used a pragmatic combination of processes for framework modification during our evaluation activities in the CAPE programme [22]. The CAPE programme included a range of interventions: seed funding for academic and policy professional collaboration in policy-focused projects, fellowships for academic placements in policy settings, or for policy professionals with HEI staff, training for policy professionals, and a range of knowledge exchange events for HEI staff and policy professionals. We modified the SPIRIT framework through iterative processes shown in Table 1, including reviews of literature; consultations with HEI staff and policy professionals across a range of policy contexts and geographic settings in England, through the CAPE programme; and piloting, refining and seeking feedback from stakeholders in academic-policy engagement.

**Table 1:** Processes to modify the SPIRIT Action Framework for academic-policy engagement interventions

Steps	Modification processes
Identifying the need and scope for framework modification	Attempting practical application of the original framework in the CAPE programme evaluation, identifying elements that did not fit or were missing Numerous meetings with the CAPE delivery team throughout the programme. Engaging with stakeholders in academia and policy to identify further missing elements.
Identifying relevant theories or models for missing elements	Targeted literature searches relating to misfitting and missing elements, particularly on behaviour change and literature on engagement actions internationally.
Combining the new elements into a modified action framework	Development of the relevant framework dimensions by integrating theories or models (i) into discrete elements and (ii) within the flow of the modified framework.
Integrating stakeholder feedback	Presentation of components of the modified framework to stakeholders in academic policy engagement (two workshops, covering broad areas of knowledge mobilisation) and academics in the field of health policy engagement (one conference paper). Comments sought on appropriateness and utility.
Piloting and refining the modified framework	Application to empirical data in the CAPE programme evaluation to assess functionality, followed by refinement of the new elements.
Testing against existing criteria for useful action frameworks	Assessment ofthe modified framework by the team, against four criteria: (i) clear purpose, (ii) informed by existing understandings, (iii) capable of guiding targeted interventions, (iv) a structure to build knowledge [20]. The manuscript including the modified framework was also reviewed by colleagues at the Sax Institute, Australia where the SPIRIT framework was originally developed.

### Findings

**Fig. 1:**
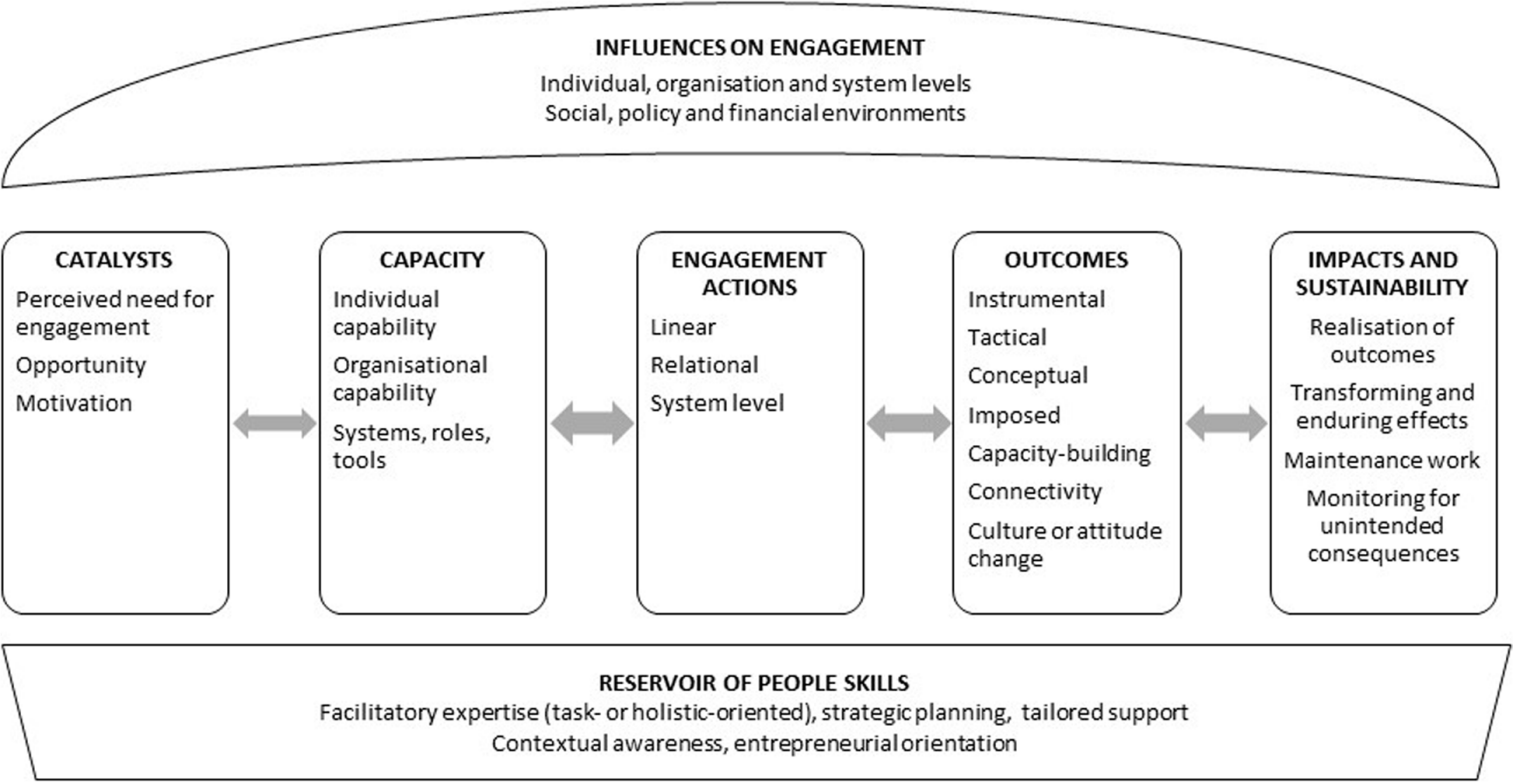
SPIRIT Action Framework Modified for Academic-Policy Engagement Interventions (SPIRIT-ME), adapted with permission from the Sax Institute. Legend: The framework acknowledges that elements in each domain may influence other elements through mechanisms of action and that these do not necessarily flow through the framework in a ‘pipeline’ sequence. Mechanisms of action are processes through which engagement strategies operate to achieve desired outcomes. They might rely on influencing factors, catalysts, an aspect of an intervention action, or a combination of elements

A number of characteristics of the original SPIRIT framework could be applied to academic-policy engagement. While keeping the core domains, we modified the framework to capture dynamics of engagement at multiple academic and policy levels (individuals, organisations and system), extending beyond the original unidirectional focus on policy agencies’ use of research. Components of the original framework, the need for modifications, and their corresponding action-oriented implications are shown in Table 2. We added a new domain, ‘Impacts and Sustainability’, to consider transforming and enduring aspects at the engagement interface. The modified action framework is shown in Fig. 1.

**Table 2:** Components of the original and modified SPIRIT action framework with corresponding action-oriented implications of the modifications

Domain	Original framework	Modified framework	Action-orientated implications
Catalysts	Policy/programme need for research New research with potential policy relevance	Need for engagement Opportunity Motivation	What prompts engagement?
Capacity	Organisation and staff value research Organisation tools and systems to support engagement actions and use of research Staff have knowledge and skills to support engagement actions and use of research	Individual capability Organisational capability Systems, roles, tools	What know how, structures and resources aid engagement?
Actions	Research engagement actions: Access research Appraise research Generate new research Interact with researchers	Academic-policy engagement actions: Linear Relational System level	What are the multi-level dynamics of the engagement?
Outcome	Research use in policymaking: Instrumental Tactical Conceptual Imposed Policy agenda setting Policy development Policy implementation Policy evaluation	Engagement outcomes: Instrumental Tactical Conceptual Imposed Capacity-building Connectivity Culture or attitude change	What does the engagement do (or not) and for whom?
Influences (contextual factors)	Policy influences	Influences at levels of individual, organisation, system Broader contexts: Social, policy and financial environments	Which contextual factors may enable or constrain engagement?
Unnamed in original [results]	Research-informed health policy and policy documents Better health system and health outcomes	Impacts and sustainability: Realisation of outcomes Transforming and enduring effects Maintenance work to sustain engagement Monitor unintended consequences	What are the lasting effects or changes and for whom? How are they recognised? How are they maintained? What was unanticipated?
Unnamed in original [prerequisites]	Reservoir of relevant and reliable research	Reservoir of people skills: Facilitatory expertise (task- or holistic-oriented) Strategic planning and support Contextual awareness Entrepreneurial orientation	What ‘hidden’ work is needed for productive engagement outcomes and impact for all involved?

#### Identifying relevant theories or models for missing elements

### Catalysts and capacity

Within our evaluation of academic-policy interventions, we identified a need to develop the original domain of catalysts beyond ‘policy/programme need for research’ and ‘new research with potential policy relevance’. Redman et al. characterised a catalyst as “a need for information to answer a particular problem in policy or program design, or to assist in supporting a case for funding” in the original framework (p. 149). We expanded this “need for information” to a perceived need for engagement, by either HEI staff or policy professionals, linking to the potential value they perceived in engaging. Specifically, there was a need to consider catalysts at the level of individual engagement, for example HEI staff wanting research to have real-world impact, or policy professionals’ desires to improve decision-making in policy, where productive interactions between academic and policy stakeholders are “necessary interim steps in the process that lead to societal impact” ( [24], p. 214). The catalyst domain expands the original emphasis on a need for research, to take account of challenges to be overcome by both the academic and policy communities in knowing how, and with whom, to engage and collaborate with [25].

We used a model proposing that there are three components for any behaviour: capability, opportunity and motivation, which is known as the COM-B model [26]. Informed by CAPE evaluation activities and our discussions with stakeholders, we mapped the opportunity and motivation constructs into the ‘catalysts’ domain of the original framework. Opportunity is an attribute of the system that can facilitate engagement. It may be a tangible factor such as the availability of seed funding, or a perceived social opportunity such as institutional support for engagement activities. Opportunity can act at the macro level of systems and organisational structures. Motivation acts at the micro level, deriving from an individual’s mental processes that stimulate and direct their behaviours; in this case, taking part in academic-policy engagement actions. The COM-B model distinguishes between reflective motivation through conscious planning and automatic motivation that may be instinctive or affective [26].

We presented an early application of the COM-B model to catalysts for engagement at an academic conference, enabling an informal exploration of attendees’ subjective views on the clarity and appropriateness, when developing the framework. This application introduces possibilities for intervention development and support by highlighting ‘opportunities’ and ‘motivations’ as key catalysts in the modified framework.

Within the ‘capacity’ domain, we retained the original levels of individuals, organisations and systems. We introduced individual capability as a construct from the COM-B model, describing knowledge, skills and abilities to generate behaviour change as a precursor of academic-policy engagement. This reframing extends the applicability to HEI staff as well as policy professionals. It brings attention to different starting conditions for individuals, such as capabilities developed through previous experience, which can link with social opportunity (for example, through training or support) as a catalyst.

### Engagement actions

We identified a need to modify the original domain ‘engagement actions’ to extend the focus beyond the use of research. We added three categories of engagement actions described by Best and Holmes [27]: linear, relational, and systems. These categories were further specified through a systematic mapping of international organisations’ academic-policy engagement activities [5]. This framework modification expands the domain to encompass: (i) linear ‘push’ of evidence from academia or ‘pull’ of evidence into policy agencies; (ii) relational approaches focused on academic-policy-maker collaboration; and (iii) systems’ strategies to facilitate engagement for example through strategic leadership, rewards or incentives [5].

### Outcomes

We retained the elements in the original framework’s ‘outcomes’ domain (instrumental, tactical, conceptual and imposed), which we found could apply to outcomes of engagement as well as research use. For example, discussions between a policy professional and a range of academics could lead to a conceptual outcome by considering an issue through different disciplinary lenses. We expanded these elements by drawing on literature on engagement outcomes [28] and through sense-checking with stakeholders in CAPE. We added capacity-building (changes to skills and expertise), connectivity (changes to the number and quality of relationships), and changes in organisational culture or attitude change towards engagement.

### Impacts and sustainability

The original framework contained endpoints described as: ‘Better health system and health outcomes’ and ‘Research-informed health policy and policy documents’. For modification beyond health contexts and to encompass broader intentions of academic-policy engagement, we replaced these elements with a new domain of ‘Impacts and sustainability’. This domain captures the continued activities required in achievement of desirable outcomes [29]. The modification allows consideration of sustainability in relation to previous stages of engagement interventions, through the identification of beneficial effects that are sustained (or not), in which ways, and for whom. Following Borst [30], we propose a shift from the expectation that ‘sustainability’ will be a fixed endpoint. Instead, we emphasise the maintenance work needed over time, to sustain productive engagement.

### Influences and facilitators

We modified the overarching ‘Policy influences’ (such as public opinion and media) in the original framework, to align with factors influencing academic-policy engagement beyond policy agencies’ use of research. We included influences at the level of the individual (for example, individual moral discretion [31]), the organisation (for example, managerial practices [31]) and the system (for example, career incentives [32]). Each of these processes takes place in the broader context of social, policy and financial environments (that is, potential sources of funding for engagement actions) [29].

We modified the domain ‘Reservoir of relevant and reliable research’ underpinning the original framework, replacing it with ‘Reservoir of people skills’, to emphasise intangible facilitatory work at the engagement interface, in place of concrete research outputs. We used the ‘Promoting Action on Research Implementation in Health Services’ (PARiHS) framework [33, 34], which gives explicit consideration to facilitation mechanisms for researchers and policy-makers [13]*.* Here, facilitation expertise includes mechanisms that focus on particular goals (task-oriented facilitation) or enable changes in ways of working (holistic-oriented facilitation). Task-orientated facilitation skills might include, for example, the provision of contacts, practical help or project management skills, while holistic-oriented facilitation involves building and sustaining partnerships or support skills’ development across a range of capabilities. These conceptualisations aligned with our consultations with facilitators of engagement in CAPE. We further extended these to include aspects identified in our evaluation activities: strategic planning, contextual awareness and entrepreneurial orientation.

#### Piloting and refining the modified framework through stakeholder engagement

We piloted an early version of the modified framework to develop a survey for all CAPE programme participants. During this pilot stage, we sought feedback from the CAPE delivery team members across HEI and policy contexts in England. CAPE delivery team members are based at five collaborating universities with partners in the Parliamentary Office for Science and Technology (POST) and Government Office for Science (GO-Science), and Nesta (a British foundation that supports innovation). The HEI members include academics and professional services knowledge mobilisation staff, responsible for leading and coordinating CAPE activities. The delivery team comprised approximately 15–20 individuals (with some fluctuations according to individual availabilities).

We assessed appropriateness and utility, refined terminology, added domain elements and explored nuances. For example, stakeholders considered the multi-layered possibilities within the domain ‘capacity’, where some HEI or policy departments may demonstrate a belief that it is important to use research in policy, but this might not be the perception of the organisation as a whole. We also sought stakeholders’ views on the utility of the new domains, for example, the identification of facilitator expertise such as acting as a knowledge broker or intermediary; providing training, advice or guidance; facilitating engagement opportunities; creating engagement programmes; and sustainability of engagement that could be conceptualised at multiple levels: personally, in processes or through systems.

#### Testing against criteria for useful action framework

The modified framework fulfils the properties of a useful action framework [20]:

1. It has a clearly articulated purpose: development and evaluation of academic-policy engagement interventions through linear, relational and/or system approaches. It has identified loci for change, at the level of the individual, the organisation or system.
2. It has been informed by existing understandings, including conceptual work of the original SPIRIT framework, conceptual models identified from the literature, published empirical findings, understandings from consultation with stakeholders, and evaluation activities in CAPE.
3. It can be applied to the development, implementation and evaluation of targeted academic-policy engagement actions, the selection of points for intervention and identification of potential outcomes, including the work of sustaining them and unanticipated consequences.
4. It provides a structure to build knowledge by guiding the generation of hypotheses about mechanisms of action in academic-policy engagement interventions, or by adapting the framework further through application in practice.

## Discussion

The proliferation of frameworks to articulate processes of research translation reveals a need for their adaptation when applied in specific contexts. The majority of models in implementation science relate to translation of research into practice. By contrast, our focus was on engagement between academia and policy. There are a growing number of academic-policy engagement interventions but a lack of published evaluations [10].

Our framework modification provides an exemplar for others who are considering how to adapt existing conceptual frameworks to address new or expanded purposes. Field et al. identified the multiple, idiosyncratic ways that the Knowledge to Action Framework has been applied in practice, demonstrating its ‘informal’ adaptability to different healthcare settings and topics [35]. Others have reported on specific processes for framework refinement or extension. Wiltsey Stirman et al. adopted a framework that characterised forms of intervention modification, using a “pragmatic, multifaceted approach” ( [36], p.2). The authors later used the modified version as a foundation to build a further framework to encompass implementation strategies in a range of settings [21]. Oiumet et al. used the approach of borrowing from a different disciplinary field for framework adaptation, by using a model of absorptive capacity from management science to develop a conceptual framework for civil servants’ absorption of research knowledge [37].

We also took the approach of “adapting the tools we think with” ( [38], p.305) during our evaluation activities on the CAPE programme. Our conceptual modifications align with the literature on motivation and entrepreneurial orientation in determining policy-makers’ and researchers’ intentions to carry out engagement in addition to ‘usual’ roles [39, 40]. Our framework offers an enabler for academic-policy engagement endeavours, by providing a structure for approaches beyond the linear transfer of information, emphasising the role of multidirectional relational activities, and the importance of their facilitation and maintenance. The framework emphasises the relationship between individuals’ and groups’ actions, and the social contexts in which these are embedded. It offers additional value by capturing the organisational and systems level factors that influence evidence-informed policymaking, incorporating the dynamic features of contexts shaping engagement and research use.

## Conclusions

Our modifications extend the original SPIRIT framework’s focus on policy agencies’ use of research, to encompass dynamic academic-policy engagement at the levels of individuals, organisations and systems. Informed by the knowledge and experiences of policy professionals, HEI staff and knowledge mobilisers, it is designed to be meaningful and accessible for people working across varied contexts and functions in the evidence-policy ecosystem. It has potential applications in how academic-policy engagement interventions might be developed, evaluated, facilitated and improved, and it fulfils Redman et al.’s criteria as a useful action framework [20].

We are testing the ‘SPIRIT-Modified for Engagement’ framework (SPIRIT-ME) through our ongoing evaluation of academic-policy engagement activities. Further empirical research is needed to explore how the framework may capture ‘additionality’, that is, to identify what is achieved through engagement actions in addition to what would have happened anyway, including long-term changes in strategic behaviours or capabilities [41–43]. Application of the modified framework in practice will highlight its strengths and limitations, to inform further iterative development and adaptation.

## Data Availability

Not applicable.
